# Focus on Vitamin D, Inflammation and Type 2 Diabetes

**DOI:** 10.3390/nu4010052

**Published:** 2012-01-20

**Authors:** Carlos Eduardo Andrade Chagas, Maria Carolina Borges, Lígia Araújo Martini, Marcelo Macedo Rogero

**Affiliations:** 1 Center for Nutrition Practice and Research, Department of Education, Institute of Biosciences, São Paulo State University, Botucatu, SP 18618-970, Brazil; Email: cchagas@ibb.unesp.br; 2 Department of Nutrition, School of Public Health, University of Sao Paulo, Sao Paulo 01246-904, Brazil; Email: mcarolborges@usp.br (M.C.B.); lmartini@usp.br (L.A.M.)

**Keywords:** vitamin D, inflammation, diabetes

## Abstract

The initial observations linking vitamin D to type 2 diabetes in humans came from studies showing that both healthy and diabetic subjects had a seasonal variation of glycemic control. Currently, there is evidence supporting that vitamin D status is important to regulate some pathways related to type 2 diabetes development. Since the activation of inflammatory pathways interferes with normal metabolism and disrupts proper insulin signaling, it is hypothesized that vitamin D could influence glucose homeostasis by modulating inflammatory response. Human studies investigating the impact of vitamin D supplementation on inflammatory biomarkers of subjects with or at high risk of developing type 2 diabetes are scarce and have generated conflicting results. Based on available clinical and epidemiological data, the positive effects of vitamin D seem to be primarily related to its action on insulin secretion and sensitivity and secondary to its action on inflammation. Future studies specifically designed to investigate the role of vitamin D on type 2 diabetes using inflammation as the main outcome are urgently needed in order to provide a more robust link between vitamin D, inflammation and type 2 diabetes.

## 1. Introduction

Type 2 diabetes is one of the main noncommunicable chronic diseases and its complications have become a major cause of morbidity and mortality worldwide. It has been estimated that 285 million individuals have diabetes, most of them type 2 diabetes [1]. Vitamin D deficiency is also considered a public health problem around the world. In 2008, it was estimated that 1 billion individuals presented vitamin D insufficiency or deficiency [2].

Much evidence suggested that vitamin D is involved in several mechanisms in addition to bone metabolism [3] and its role in abnormal glucose metabolism as well as in type 2 diabetes has been demonstrated [4,5]. A recent review indicates that vitamin D deficiency may predispose to glucose intolerance, altered insulin secretion and type 2 diabetes [6], either through a direct action via vitamin D receptor (VDR) activation or indirectly via calcemic hormones and also via inflammation [7]. 

Furthermore, in observational studies the risk of diabetes was negatively associated with increased vitamin D concentrations [6,8]. In fact, Mitri *et al.* [9], in a systematic review, confirmed such evidence by evaluating vitamin D intake and 25-hydroxyvitamin D (25OHD) levels. In 8 observational studies, vitamin D intake >500 international units (IU)/day decreased the risk of type 2 diabetes by 13% compared with vitamin D intake <200 IU/day. Individuals with the highest 25OHD status (>25 ng/mL) had a 43% lower risk of developing type 2 diabetes (95% confidence interval 24–57%) compared with those in the lowest group (<14 ng/mL). 

On the other hand, information pooled from vitamin D intervention trials lack conclusive evidence. In the same systematic review [9], no effect of vitamin D supplementation on glycemic outcomes were demonstrated in *post hoc* analysis from eleven trials. However, it has been observed some potential benefits of vitamin D supplementation in non-diabetics [10]. There are several potential reasons for the conflicting findings from human studies of vitamin D and diabetes, which are discussed in the present review.

Inflammation participates in host defenses against infectious agents and injury, but it also contributes to the pathophysiology of many chronic diseases. There is evidence for a direct link between type 2 diabetes and subclinical inflammation, which supports the concept that such disease is, at least in part, an inflammatory condition [11]. Moreover, it has been observed that the relationship between vitamin D and low-intensity chronic inflammation and insulin resistance in type 2 diabetes can be mediated in part by the immune-modulating properties of the 1,25(OH)_2_D_3_, which is able to downregulate the production of pro-inflammatory cytokines [12].

Considering that inflammatory status as well vitamin D insufficiency create an environment conducive to the development and progression of several diseases, the present review will focus on the associations observed between vitamin D status and its potential immune-modulating effects in the metabolism of type 2 diabetes biomarkers. 

## 2. Inflammation, Insulin Resistance and Type 2 Diabetes

Chronic low-grade inflammation, frequently observed in obese individuals, is involved in the development of insulin resistance, which increases the risk of type 2 diabetes. The first link between obesity, inflammation and insulin action came from a study developed by Hotamisligil *et al.* [13], which demonstrated that tumor necrosis factor (TNF)-α mRNA expression in the adipose tissue of obese animal (fa/fa rat and ob/ob mouse) was increased and that the neutralization of TNF-α improved insulin action on glucose uptake. It is now acknowledged that not only TNF-α but an array of inflammatory cytokines are elevated in obese tissues, including interleukin (IL)-1β, IL-6, monocyte chemoattractant protein (MCP)-1, and others [14]. A major finding advancing in the understanding of obesity-induced inflammation was the discovery that immune cells, in particular adipose tissue infiltrated macrophages, largely contribute to the increased production of inflammatory mediators [15,16].

There is strong evidence that activation of inflammatory pathways interferes with normal metabolism and disrupts proper insulin signalling [17]. Briefly, insulin binding to its receptor triggers tyrosine phosphorylation of insulin receptor substrates (IRS), leading to activation of phosphatidylinositol 3-kinase (PI3K)-Akt pathway, which is responsible for insulin action on glucose uptake and suppression of gluconeogenesis [18]. In response to inflammatory signals, c-jun *N*-terminal kinase (JNK) and inhibitor of κB kinase (IKK) are activated and can target IRS-1 for serine phosphorylation, which inhibits the insulin receptor signalling cascade. Not only JNK and IKK, but also other kinases, such as protein kinase C (PKC)-θ, can inhibit IRS-1 through serine phosphorylation, implying that activation of diverse cellular networks can antagonize insulin signalling. Apart from inhibiting insulin action through targeting insulin signalling molecules, JNK and IKK can also regulate downstream transcriptional programs through the transcription factors activator protein (AP)-1 and nuclear factor (NF)-κB, respectively, resulting in increased expression of proinflammatory cytokines, such as IL-1β and TNF-α [17,19,20]. These cytokines can target cell membrane receptors, feeding into inflammatory response and exacerbating insulin resistance. Another important molecular mediator that link proinflammatory cytokine to inhibition of insulin signalling are suppressors of cytokine signalling (SOCS) 1 and 3, induced by IL-6, which lead to ubiquitinylation and degradation of IRS proteins [21]. 

Insulin resistance can also be triggered by the presence of metabolic stressors, such as high blood non-esterified fatty acids (NEFA) levels, which compromises insulin sensitivity by reducing the action of this hormone in peripheral tissues, such as the liver, skeletal muscle and adipose tissue [22,23]. Another systemic factor influencing insulin sensitivity is adiponectin. Plasma adiponectin levels in humans are negatively correlated with fasting insulin concentrations and positively correlated with insulin sensitivity, suggesting that the hormone is able to sensitize peripheral tissues to insulin action. However, certain inflammatory mediators, such as TNF-a and IL-6, which have been shown to be elevated in obese and insulin resistant individuals, are inhibitors of adipose tissue adiponectin mRNA expression and protein secretion [24]. In addition, adiponectin impairs the production of proinflammatory cytokines, such as TNF-a and interferon-g (IFN-g), in human macrophages and reduces their phagocytic capacity while inducing the production of the anti-inflammatory mediators IL-10 and IL-1 receptor antagonist (IL-1RA) by human monocytes, monocyte-derived macrophages and dendritic cells [25].

## 3. Vitamin D and Inflammation

**Figure 1:**
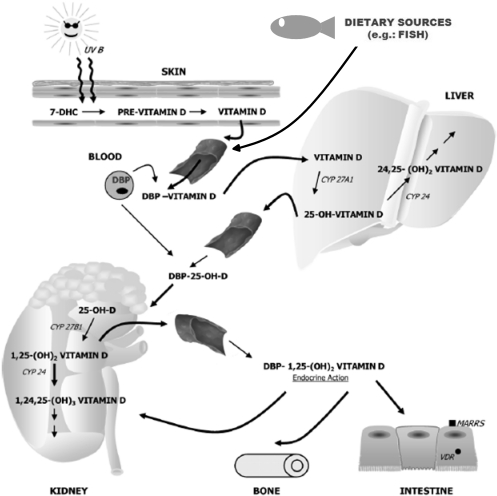
Cutaneous synthesis and metabolism of vitamin D. In the skin, 7-dehydrocholesterol (DHC) can be converted to pre-vitamin D in response to ultraviolet B (UVB) radiation from the sun. Pre-vitamin D is then converted to vitamin D. Continued cutaneous exposure to UVB can produce various photoproducts (not shown) from both pre-vitamin D and vitamin D. Vitamin D (and other vitamin D metabolites) are carried in the blood by a 50-kD vitamin D-binding protein (DBP). Vitamin D is converted in the liver by the P450 enzyme CYP27A1 to 25-hydroxyvitamin D (25OHD), which is the major form of vitamin D found in the blood. In the kidney, another P450 enzyme, CYP27B1, adds a hydroxyl group at the C-1 position of 25OHD to form the active vitamin D hormone 1,25-dihydroxyvitamin D, or 1,25(OH)_2_D_3_. Both 25OHD and 1,25(OH)_2_D_3_ are hydroxylated at C-24 by CYP24, which initiates their inactivation and metabolic breakdown. Vitamin D receptor (VDR)-mediated gene expression in response to 1,25(OH)_2_D_3_ occurs in many different tissues, including classical vitamin D target organs such as intestine, bone, and kidney. The active vitamin D hormone can also stimulate very rapid changes at the plasma membrane that are mediated by a 1,25(OH)_2_D_3_membrane-associated rapid response steroid hormone binding protein (MARRS). Adapted from Martini and Wood [26].

During the exposure to sunlight, ultraviolet B (UVB) photons penetrate into the skin and are absorbed by 7-dehydrocholesterol inducing the formation of previtamin D (Figure 1). This is an unstable form of vitamin D that rapidly undergoes rearrangement to form vitamin D_3_ (cholecalciferol). Vitamin D_2_ (ergocalciferol) is the form of vitamin D that occurs in plants and is used to fortify certain foods, such as fluid milk. Both vitamin D forms eventually enter the circulation bound to a vitamin D binding protein and are metabolized in the liver by the vitamin D-25-hydroxylase enzyme (25-OHase or CYP27A1) to 25-hydroxyvitamin D (calcidiol), the main vitamin D form circulating in plasma and a substrate for production of the hormonally active metabolite 1,25-dihydroxyvitamin D, 1,25(OH)_2_D_3_ (calcitriol) [26].

It is recognized that VDR, the receptor of the steroid hormone 1,25(OH)_2_D_3_, is widely distributed in more than 38 tissues, where it clearly controls vital genes related to bone metabolism, oxidative damage, chronic diseases and inflammation [27].

VDR is constitutively expressed by macrophages and dendritic cells, which suggests that vitamin D plays an important role in the modulation of inflammatory response. The 1,25(OH)_2_D_3_ can be synthesized by both cell types, since they express the enzymes 25-hydroxylase and 1α-hydroxylase, which enables the production of 25OHD and 1,25(OH)_2_D_3_, respectively [28,29]. In macrophages and dendritic cells, the enzyme 1α-hydroxylase is predominantly regulated by inflammatory mediators, such as interferon (IFN)-γ and lipopolysaccharides (LPS) [30]. 

**Figure 2:**
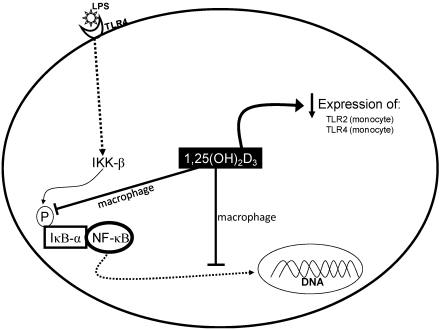
Vitamin D modulates the inflammatory response of immune cells, such as macrophages and monocytes. Adapted from Borges *et al.* [37] (IKK: IκB kinase; IκB: inhibitor of NF-kB; LPS: lipopolysaccharides; TLR: Toll-like receptor).

Macrophages are cells with a large capacity for cytokine production, in particular TNF-α, which is one of the most important products released from these cells [31]. Transcriptional activation of the TNF-α gene in macrophages is largely dependent on the NF-κB-dependent transcriptional activation, which is a major regulator of immune, inflammatory and stress responses [32]. In LPS-stimulated murine macrophages, 1,25(OH)_2_D_3_ up-regulates the inhibitor of NF-κB (IκB-α) by increasing mRNA stability and decreasing IkB-α phosphorylation. The increase in IκB-α levels leads to a reduction in nuclear translocation of NF-κB, thereby causing a decline in activity. In view of the key role of NF-κB as transcription factor of inflammatory mediators, it should be suggested that 1,25(OH)_2_D_3_ has anti-inflammatory action in macrophages [33]. Furthermore, 1,25(OH)_2_D_3_ suppresses the expression of TLR2 and TLR4 protein and mRNA in human monocytes in a time- and dose-dependent fashion (Figure 2) [34]. Incubation of isolated monocytes with 1,25(OH)_2_D_3_ attenuates the expression of proinflammatory cytokines involved in insulin resistance such as IL-1, IL-6 and TNF-alpha in type 2 diabetic patients [35]. This fact may be related to the downregulation of NF-κB activity, as suggested by studies with P388D1 cells, a murine macrophage-like cell line [33,36].

Despite the fact that experimental data support the involvement of vitamin D in modulating the inflammatory response, clinical and epidemiological studies are still scarce. Observational studies have generated conflicting results. Some cross-sectional studies indicate that hypovitaminosis D is associated with higher serum levels of inflammatory biomarkers, such as IL-6, TNF-α, and C-reactive protein (CRP), in healthy [38,39,40,41] and in obese subjects [42], while others could not confirm these findings [43,44,45,46]. Given the observational design, cross-sectional studies cannot prove causality or fully discount residual confounding by unmeasured variations. In this context, randomized placebo-controlled clinical trials are extremely useful to address the hypothesis that hypovitaminosis D might induce a higher inflammatory response. Table 1 presents clinical trials investigating the effect of vitamin D supplementation on serum inflammatory biomarkers. In some diseases associated with inflammation, such as chronic heart failure, chronic kidney disease and osteoporosis, vitamin D supplementation seems to attenuate serum TNF-α levels and to increase serum IL-10 concentration [47,48,49]. In a study involving subjects with either normal or impaired fasting glucose, the combined calcium-vitamin D supplementation (500 mg calcium + 700 IU cholecalciferol per day) for 3 years did not influence systemic CRP or IL-6 [50]. On the other hand, healthy overweight subjects participating in a weight-reduction program, when supplemented with vitamin D (3332 IU cholecalciferol/day for twelve months), experienced a greater decrease in serum TNF-α levels, but not in IL-6 and CRP concentration, when compared to placebo group [51]. 

**Table 1:** Clinical trials investigating the effect of vitamin D supplementation on serum inflammatory biomarkers.

Ref.	Number and characteristics of subjects	Intervention and duration	Vitamin D effect on inflammatory serum biomarkers
[10]	81 South Asian women with insulin resistance. Median serum 25OHD at baseline: 21 nmol/L.	100 μg of vitamin D_3_ or placebo for 6 months.	No effect on C-reactive protein.
[47]	123 patients with congestive heart failure. Mean serum 25OHD at the baseline: 36 nmol/L.	Oral supplementation (50 μg/day vitamin D_3_ plus 500 mg of calcium) for 9 months.	No differences in TNF-α and C-reactive protein. Significant increase in interleukin 10.
[48]	34 haemodialysis patients. Mean serum 25OHD at baseline: not reported.	Oral (0.5 μg/day; *n* = 18) or intravenous (1 μg 3× week; *n* = 16) calcitriol for 6 months.	Oral calcitriol: No differences in TNF-α, interleukin 1 and interleukin 6;
			Intravenous calcitriol: significant decrease in TNF-α, interleukin 1 and interleukin 6.
[49]	70 post-menopausal women with osteoporosis. Mean serum 25OHD at baseline: not reported.	0.5 μg/day of calcitriol and 1,000 mg/day of calcium or placebo (only 1,000 mg/day of calcium) for 6 months.	Significant decrease in decrease in TNF-α and interleukin 1. No differences in interleukin 6.
[50]	222 non-obese subjects with normal fasting glucose and 92 non-obese with impaired fasting glucose. Mean serum 25OHD at baseline in both groups: 76 nmol/L.	700 IU of vitamin D_3_ or placebos for 3 years.	No differences in C-reactive protein and interleukin 6.
[51]	200 healthy overweight subjects. Mean serum 25OHD at baseline: 30 nmol/L.	83 μg/day of vitamin D_3 _or placebo in a double-blind manner for 1 year while participation in a weight-reduction program.	More pronounced decrease in TNF-α in vitamin D group than in placebo group.
[52]	218 long-term inpatients. Mean serum 25OHD at baseline: 23 nmol/L.	0, 400 or 1200 IU/day of vitamin D_3_ for 6 months.	No differences in C-reactive protein.
[53]	125 haemodialysis patients. Mean serum 25OHD at baseline: 32 nmol/L.	100,000 IU/month of vitamin D_3_ for 15 months.	No differences in C-reactive protein.
[54]	158 haemodialysis patients. Thirty-nine had diabetes and 54 had hypertension. Mean serum 25OHD at baseline: 55.75 nmol/L.	Vitamin D_3_ for 6 months according to 25OHD serum levels at the baseline:	Significant decrease in C-reactive protein.
		- 50,000 IU/week for those with 25OHD serum levels < 15 ng/mL;	
		- 10,000 IU/week for those with 25OHD between 16 and 30 ng/mL;	
		- 2,700 IU 3x week for those with 25OHD > 30 ng/mL.	
[55]	30 haemodialysis patients. Mean serum 25OHD at baseline: 45.5 nmol/L.	Weekly supplementation of vitamin D_3_ for 24 weeks: 50,000 IU in the first 12 weeks and 20,000 IU in the last 12 weeks.	Significant decrease in C-reactive protein and interleukin 6.

## 4. Vitamin D and Type 2 Diabetes

In contrast to type 1 diabetes, which is related to autoimmune destruction of pancreatic β cells, leading to absolute insulin deficiency, type 2 diabetes development involves impaired pancreatic β cell function, insulin resistance and inflammation. Although mechanistically unclear, it has been suggested that both environmental and genetic factors seem to be involved in type 2 diabetes development [56]; also, human and experimental data support the role of vitamin D on these pathways [8,57]. 

Due to the presence of both 1-α-hydroxylase and VDR in pancreatic β cells, vitamin D is important for insulin synthesis and release [8,56]. In rats, vitamin D deficiency induced impairment of insulin secretion and glucose tolerance that was partially corrected after vitamin D replenishment [58,59]. Moreover, vitamin D is also involved in insulin sensitivity by controlling calcium flux through the membrane in both β cells and peripheral insulin-target tissues [57]. 

The initial observations linking vitamin D to type 2 diabetes in humans came from studies showing that both healthy and diabetic subjects had a seasonal variation of glycemic control [60,61]. Since then, several recent human studies have associated vitamin D status with type 2 diabetes development (Table 2). It should be highlighted that after statistical adjustments for potential risk factors of type 2 diabetes, such as body mass index, the association between vitamin D and type 2 diabetes was attenuated in one study [62] and no longer significant in another one [63]. Almost all studies used serum 25OHD as a biomarker for vitamin D stores, while studies investigating vitamin D intake are scarce.

**Table 2:** Human studies that associate vitamin D with type 2 diabetes.

Ref.	Study design	Subjects included	Main outcome
[62]	Cohort (Mini-Finland Health Survey)	4097 individuals followed-up for 17 years.	The highest *versus* the lowest serum 25OHD: RR = 0.70; 95% CI = 0.42–1.16); *p* for trend = 0.07).
[63]	Cohort (Tromsø Study)	4157 non-smokers and 1962 smokers followed-up for 11 years.	Baseline serum 25OHD was inversely associated with type 2 diabetes.
[64]	Cohort (Nurses’ Health Study)	83,779 women followed-up for 20 years.	The highest *versus* the lowest category of vitamin D intake from supplements: RR = 0.87; 95% CI = 0.75–1.00; *p* for trend = 0.004).
[65]	Nested case-control	412 cases and 986 controls.	The highest *versus* the lowest quartiles of serum 25OHD: OR = 0.28 (95% CI = 0.10–0.81) in men and OR = 1.14 (95% CI = 0.60–2.17) in women.
[66]	Meta-analysis	Polled data from 2 cohorts studies with 8627 individuals aged 40–79 years.	The highest *versus* the lowest serum 25OHD: RR = 0.66; 95% CI = 0.50–0.87.
[67]	Cohort (Framingham Study)	3066 (1402 men and 1664 women) followed-up for 7 years.	A higher 25OHD serum levels is associated with decreased risk of type 2 diabetes.
[6]	Nested case-control	608 cases and 559 controls.	The highest *versus* the lowest serum 25OHD quartile: OR = 0.52; 95% CI = 0.33–0.83.
[68]	Cross-sectional	210 individual aged more than 40.	Vitamin D deficiency was more common in diabetic compared to control.
[69]	Cross-sectional	668 individuals aged 70–74 years.	Serum 25OHD < 50 nmol/L doubled the risk of newly diagnosed type 2 diabetes.
[70]	Cohort (AusDiab study)	5200 individuals; mean age 51 years.	Each 25 nmol/L increment in serum 25OHD was associated with a 24% reduced risk of type 2 diabetes (OR = 0.76; 95% CI = 0.63–0.92).
[71]	Cross-sectional	2465 subjects.	Serum 25OHD ≥ 80 nmol/L *versus* ≤37 nmol/L in Caucasians: OR = 0.5; 95% CI = 0.1–0.7.
[9]	Systematic review of 7 observational cohort studies.	238,424 individuals aged 30–75 years.	Vitamin D intake >500 *versus* <200 UI: risk of type 2 diabetes 13% lower. Serum 25OHD level (>25 ng/mL *versus* <14 ng/mL): risk of type 2 diabetes 43% lower.

Based on data from epidemiological studies, vitamin D supplementation is considered a potential and inexpensive therapy not only to decrease the risk, but also to improve glycemic parameters in type 2 diabetic patients [56]. In subjects at high risk of type 2 diabetes and with baseline serum 25OHD level of 26.5 nmol/L, vitamin D supplementation (2000 UI once daily) was associated with improved β cell function in adults [72]. Daily intake of vitamin D-fortified yogurt (either with or without added calcium) improved serum 25OHD levels and glycemic status in type 2 diabetic patients with baseline 25OHD serum level of 44.5 nmol/L. In the same study, an inverse correlation between changes in serum 25OHD and fasting serum glucose and homeostasis model assessment of insulin resistance (HOMA-IR) was observed [73]. In a randomized, controlled, double-blinded intervention study, insulin resistant and vitamin D deficient (serum 25OHD < 50 nmol/L) subjects supplemented with vitamin D (4000 UI, daily, for 6 months) had improved serum 25OHD level, insulin sensitivity and insulin resistance when compared to controls, while no effects were observed on lipid profile, C-reactive protein and insulin secretion [10]. Similarly, in another randomized controlled trial, type 2 diabetes patients with baseline serum 25OHD concentration <50 nmol/L treated with a single dose of vitamin D (100,000 or 200,000 UI) had lower systolic blood pressure than controls, but HOMA-IR was significantly improved only in subjects who received the highest dose [74]. 

It is important to notice that in a meta-analysis by Pittas *et al.* [75], among six intervention trials reviewed (five with vitamin D alone and one with calcium and vitamin D), none were able to elicit a remarkable change in measures of glucose intolerance. In 2010, the same group of investigators revisited the question of vitamin D supplementation and plasma glucose [76]. From the randomized controlled trials included, three of them used vitamin D alone, and again no convincing evidence that vitamin D supplementation have benefits on blood glucose control was observed. However, not all studies reported 25OHD at the baseline and studies vary in the amount of vitamin D, type of vitamin D, length of supplementation, number of subjects, and subject characteristics such as non-diabetic, diabetics, healthy, overweight, insulin resistant, and gender. Thus, it would be important that all future trials describe 25OHD serum levels at the baseline and investigate not only blood glucose but also the role of vitamin D on glucose tolerance, insulin secretion, insulin sensitivity and ultimately with incident of type 2 diabetes. In order to provide more robust evidences on vitamin D in both prevention and management of type 2 diabetes, future trials should also investigate the role of higher doses of vitamin D supplementation in larger populations and for longer periods. 

Despite the lack of consensus regarding the adequate 25OHD serum levels to prevent and improve glycemic parameters in type 2 diabetes patients, it should be highlighted that the positive effect of vitamin D supplementation was observed when baseline 25OHD serum levels improved to near 80 nmol/L after the intervention [10,73,74]. Accordingly, data from a cross-section study described that subjects who had 25OHD serum levels ≥80 nmol/L had decreased risk of developing type 2 diabetes when compared to the ones who had ≤37 nmol/L [71]. 

Although not uniformly, it was suggested that several genetic polymorphisms in genes related to vitamin D metabolism, such as DBP and VDR, may predispose subjects to type 2 diabetes [56]. Three variants of DBP gene (Gc1f, Gc1s, and Gc2) were associated with differences in oral glucose tolerance in nondiabetic Pima Indians [77]. Although no difference was observed between DBP genotypes regarding plasma glucose concentration, normal glucose tolerance Japanese subjects with Gc1s-2 and 1s-1s genotypes had significantly higher fasting plasma insulin concentration and HOMA-IR than the ones with 1f-1f [78]. However, DBP gene polymorphism was not associated with diabetes in while Americans of European origin [79] and in French Caucasians [80]. 

A similar scenario is observed for VDR genotype. Regarding VDR ApaI polymorphisms, older adults without diabetes that have aa genotype had higher fasting plasma glucose and prevalence of glucose intolerance than those with AA genotype. In the same study, bb genotype of VDR BsmI polymorphism was associated with insulin resistance in subjects with diabetes [81]. VDR polymorphisms were also associated with type 2 diabetes in two Indian case-control studies [82,83]. However, in Polish [84] and Turkish [85] case-control studies, no differences were observed between groups regarding allele frequency of VDR polymorphisms.

## 5. Conclusion

In summary, there is consistent evidence supporting that vitamin D status is related to and is important to regulate some pathways related to type 2 diabetes development. Although experimental studies support the involvement of vitamin D in modulating the inflammatory response, human studies investigating inflammatory biomarkers specifically in subjects with or at high risk of developing type 2 diabetes are scarce. Thus, based on available clinical and epidemiological data, the positive effects of vitamin D seem to be primarily related to its action on insulin secretion and sensitivity and secondary to its action on inflammation. Future studies specifically designed to investigate the role of vitamin D on type 2 diabetes using inflammation as the main outcome are urgently needed in order to provide a more robust link between vitamin D, inflammation and type 2 diabetes. Furthermore, genetic polymorphisms studies are also important in order to identify groups that are more susceptible to vitamin D deficiency and to developing type 2 diabetes in the population. 
